# Oxygenation of Earth’s atmosphere induced metabolic and ecologic transformations recorded in the Lomagundi-Jatuli carbon isotopic excursion

**DOI:** 10.1128/aem.00093-24

**Published:** 2024-05-31

**Authors:** Dawn Y. Sumner

**Affiliations:** 1 Department of Earth and Planetary Sciences, University of California, Davis, Davis, California, USA; 2 Microbiology Graduate Group, University of California, Davis, Davis, California, USA; 3 Feminist Research Institute, University of California, Davis, Davis, California, USA; University of Wisconsin-Madison, Madison, Wisconsin, USA

**Keywords:** microbial ecology, Paleoproterozoic, metabolism, carbonate geochemistry, deep evolution, remineralization

## Abstract

The oxygenation of Earth’s atmosphere represents the quintessential transformation of a planetary surface by microbial processes. In turn, atmospheric oxygenation transformed metabolic evolution; molecular clock models indicate the diversification and ecological expansion of respiratory metabolisms in the several hundred million years following atmospheric oxygenation. Across this same interval, the geological record preserves ^13^C enrichment in some carbonate rocks, called the Lomagundi-Jatuli excursion (LJE). By combining data from geologic and genomic records, a self-consistent metabolic evolution model emerges for the LJE. First, fermentation and methanogenesis were major processes remineralizing organic carbon before atmospheric oxygenation. Once an ozone layer formed, shallow water and exposed environments were shielded from UVB/C radiation, allowing the expansion of cyanobacterial primary productivity. High primary productivity and methanogenesis led to preferential removal of ^12^C into organic carbon and CH_4_. Extreme and variable ^13^C enrichments in carbonates were caused by ^13^C-depleted CH_4_ loss to the atmosphere. Through time, aerobic respiration diversified and became ecologically widespread, as did other new metabolisms. Respiration displaced fermentation and methanogenesis as the dominant organic matter remineralization processes. As CH_4_ loss slowed, dissolved inorganic carbon in shallow environments was no longer highly ^13^C enriched. Thus, the loss of extreme ^13^C enrichments in carbonates marks the establishment of a new microbial mat ecosystem structure, one dominated by respiratory processes distributed along steep redox gradients. These gradients allowed the exchange of metabolic by-products among metabolically diverse organisms, providing novel metabolic opportunities. Thus, the microbially induced oxygenation of Earth’s atmosphere led to the transformation of microbial ecosystems, an archetypal example of planetary microbiology.

**IMPORTANCE:**

The oxygenation of Earth’s atmosphere represents the most extensive known chemical transformation of a planetary surface by microbial processes. In turn, atmospheric oxygenation transformed metabolic evolution by providing oxidants independent of the sites of photosynthesis. Thus, the evolutionary changes during this interval and their effects on planetary-scale biogeochemical cycles are fundamental to our understanding of the interdependencies among genomes, organisms, ecosystems, elemental cycles, and Earth’s surface chemistry through time.

## INTRODUCTION

Earth and life have co-evolved over billions of years. Interdependencies among environments, ecosystems, organisms, and genomes promote this co-evolution, simultaneously transforming life and Earth’s surface through metabolic activities and interactions. Some of the most significant biogeochemical transformations were associated with the production of O_2_ from photosynthesis. Molecular oxygen transformed habitable environments in response to shifting geological boundary conditions; its accumulation affected the biosphere at all scales from mutations to ecosystem structure. These transformations are partially preserved in both the geological record and the genomes of extant organisms. Reconstructing the history and consequences of this planetary-scale, microbially induced redox change requires integrating clues from these disparate records into self-consistent explanatory models that make predictions that can be tested by data from both types of records. Sufficient clues are now available to make this possible for the aftermath of the oxygenation of Earth’s atmosphere.

In the geological record, geochemical signatures like the carbon isotopic composition of carbonates and organic matter record ecosystem and metabolic processes. Carbon-based molecules and reactions are the foundation of biological activity, and changes in carbon isotope ratios have been used extensively to interpret biosphere processes [e.g., references (1–3)]. In general, primary productivity preferentially incorporates ^12^C over heavier ^13^C atoms. This leads to a depletion of ^13^C in organic matter and enrichment in the remaining dissolved inorganic carbon (DIC) in the environment. The long-term balance in ^13^C to ^12^C ratios in DIC in the oceans depends on inputs of carbon from Earth’s interior and weathering of rocks, the burial of organic matter, and the burial of carbonate rocks, which form from environmental DIC, recording its isotopic composition [e.g., reference (3)]. In general, the more organic carbon that gets buried relative to other processes, the higher the enrichment of ^13^C in DIC and thus carbonate rocks. These signatures can be measured in the rock record and are interpreted as reflecting local, regional, or global processes, depending on their patterns and context. The global burial of organic carbon is also tied to the oxidation state of the Earth’s surface; high net burial rates of reduced organic carbon increase the oxidation state of the Earth’s surface [e.g., reference (3)]. Thus, carbon isotopic values and the oxidation state of Earth’s surface are commonly co-interpreted to understand global biogeochemical processes.

Sometimes, biosphere processes create DIC carbon isotopic variations that are challenging to understand without also considering interdependencies among contemporaneous evolutionary and environmental changes. One of these examples is the global ~2.3–2.1 Ga Lomagundi-Jatuli carbon isotopic excursion (LJE), which followed the oxygenation of Earth’s atmosphere (3–10). This excursion is characterized by extreme ^13^C enrichment in carbonates, which has been traditionally interpreted as due to enhanced organic carbon burial [(3) and references therein (5, 8)]. Enhanced organic carbon burial should lead to an increase in oxidation state for the oceans and atmosphere [e.g., reference (8)]. However, the LJE postdates oxygenation of the atmosphere rather than predating or being contemporaneous with it, and organic-rich rocks are not particularly abundant during this interval (3, 6, 8–10). In addition, the observed ^13^C enrichment of carbonate rocks varies significantly with their depositional environment. The extreme enrichments are mostly present in the shallowest water environments with most of the deeper marine carbonates having ^13^C to ^12^C ratios typical of most of Earth’s history (6, 9, 10). Thus, the details in the timing and patterns of ^13^C enrichments are not consistent with a traditional interpretation of globally enhanced organic carbon burial causing the LJE and oxygenation of the atmosphere (3, 10). Some researchers have called on local processes to cause local ^13^C enrichments in DIC, for example, microbial methanogenesis with the loss of methane to the atmosphere or alteration of the carbonate isotopic signatures [e.g., references (1, 6, 9–12)]. However, these local models do not provide mechanisms for the global-scale initiation and end of the LJE; the conditions for these enrichments have existed locally throughout Earth’s history, including in modern environments [see Discussion in reference (3)]. If methanogenesis or carbonate alteration were the causes of the LJE, additional mechanisms are necessary for making these local conditions particularly abundant in the 100–250 million years after oxygenation of the atmosphere.

In this contribution, I present a novel conceptual metabolism evolution model that explains the initiation of the LJE as the proliferation of cyanobacterial primary productivity in shallow environments after the formation of an ozone layer. This expansion in primary productivity was followed by the evolution and diversification of new respiratory metabolisms that led to a transformation in organic carbon remineralization and eventually to the end of the LJE. This explanatory model is consistent with the patterns in both the geologic and genomic records and provides testable predictions for future work.

### The Lomagundi-Jatuli excursion

The LJE is defined as a 100–250 million year long interval that is characterized by unusually high carbonate carbon isotope values, commonly between +5‰ and +10‰ with some up to +30‰ [δ^13^C_carb_, where δ^13^C ={[(^13^C/^12^C)_sample_/(^13^C/^12^C)_standard_] − 1} × 1,000, all values reported relative to the VPDB standard; (3–10, 13)]. Its absolute age and duration are poorly constrained due to challenges in dating the near-shore carbonate rocks that preserve the excursion. However, it started after evidence of oxygenation of the atmosphere within the same sequences, giving an initiation age of roughly 2.3 Ga and likely ended around 2.1 Ga, but data in hand do not require a globally synchronous initiation nor termination for the LJE [see Discussion and Fig. 8 in reference (3)]. These characteristics make the excursion exceptional and difficult to interpret both with respect to its duration and its evidence for extremely ^13^C-enriched DIC (δ^13^C_DIC_). In addition, isotopic variations within the LJE are unusual. δ^13^C_carb_ values tend to have two peaks, one centered at 0‰ and one centered at +7‰ (3, 10). In addition, carbon isotopic curves for any given sequence usually lack the typical rising or falling isotopic trends characteristic of most carbon isotopic excursions that reflect global changes in the rates of organic carbon burial (3). Rather, “normal” values near 0‰ and ^13^C-enriched values are intermixed or vary with depositional environment, suggesting that δ^13^C_carb_ values do not reflect changes to oceanic δ^13^C_DIC_ (3, 4, 6, 10, 13).

Studies of organic carbon isotopic values (δ^13^C_org_) through the LJE are sparse [but see references (5, 8, 9)], in part because the shallow water carbonates hosting the LJE isotopic excursion usually contain little organic carbon. For those sections with organic matter hosted in ^13^C-enriched shallow water carbonates, δ^13^C_org_ values are either typical of cyanobacterial photosynthesis from a “normal” DIC pool [−25‰ to −30‰; (9)] or are enriched in ^13^C consistent with primary productivity from a DIC pool enriched in ^13^C [−14‰ to −25‰; (8)]. Organic carbon associated with deeper water deltaic shales that accumulated within the LJE interval has values from −20‰ to −47‰ (8, 9). Some of the heavier values are consistent with primary productivity from seawater with δ^13^C_DIC_ of approximately 0‰, but those below −30‰ likely require methanotrophy to provide a significant proportion of the preserved organic matter (8, 9). Given the large number of processes that can influence δ^13^C_org_, distinguishing among them with sparse data is challenging, and insufficient constraints make the data difficult to use for testing specific models.

The long duration, extreme δ^13^C_carb_ values, and variations in δ^13^C_carb_ with the depositional environment make the LJE dissimilar to most other global isotopic excursions that can be interpreted as intervals of enhanced organic carbon burial (1, 3, 10, 12). Specifically, variability within the LJE suggests that local processes influenced the δ^13^C_DIC_ of specific environments (3, 6, 7, 9–12) even though it is expressed globally. Local DIC can become enriched in ^13^C due to microbial ecosystem processes. Specifically, methanogenesis can lead to very high local δ^13^C_DIC_, sometimes exceeding +10‰ (11, 12). Others have proposed that methanogenesis explains the high δ^13^C_carb_ values in the LJE interval, whether methanogenesis occurred within the sediment or with CH_4_ loss to the atmosphere (1, 11, 12). However, these prior models have not provided a mechanism for explaining the global initiation of the LJE or its termination if local processes are causing the extreme ^13^C enrichments. In this contribution, I propose that ecological and evolutionary responses to the oxygenation of the atmosphere provide mechanisms for the initiation and termination of the LJE that are consistent with the isotopic record as well as molecular clock models based on the analyses of extant genomes.

### Organic matter remineralization and metabolic evolution

**Fig 1:**
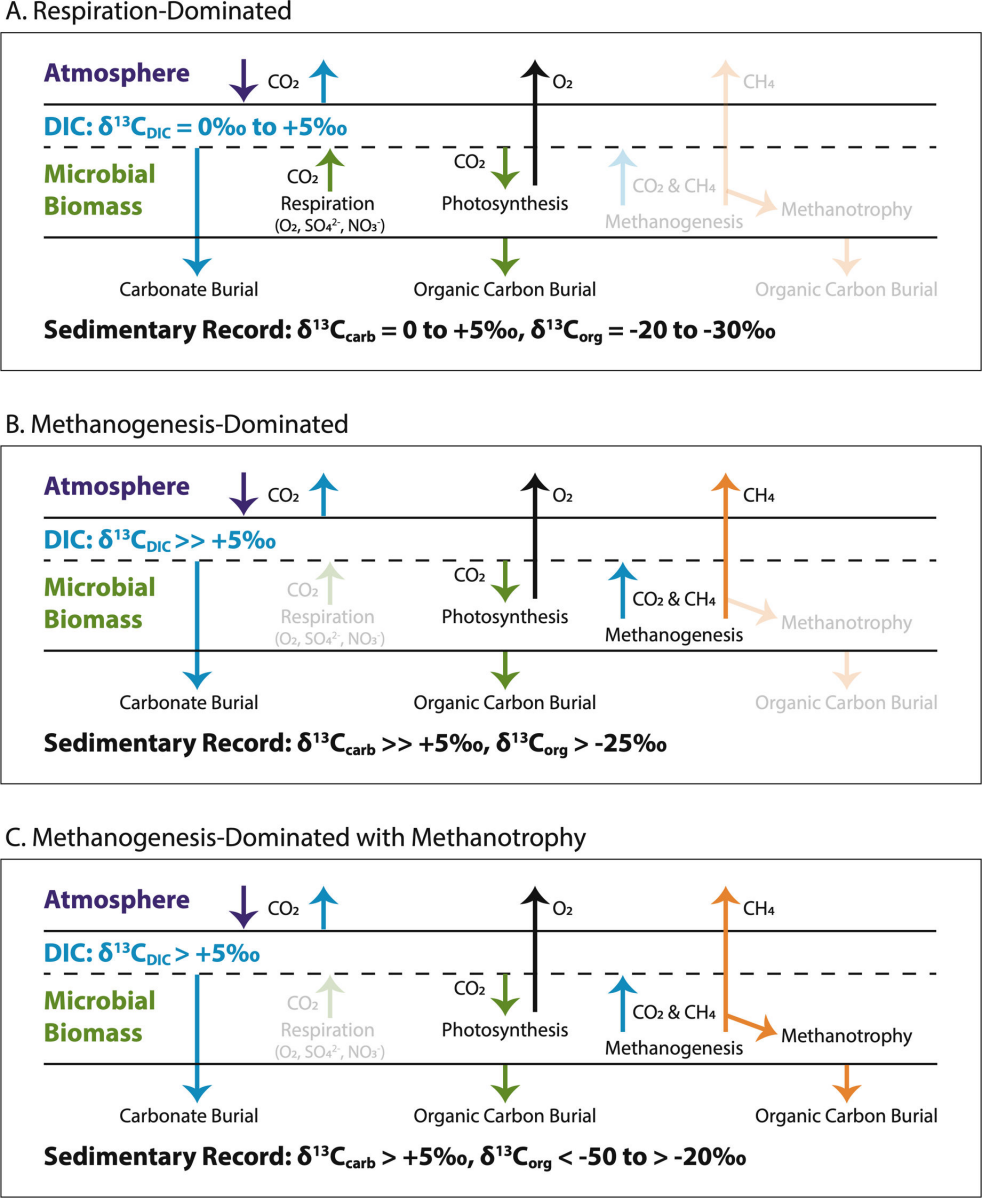
Local carbon cycle models for respiration-dominated (**A**), methanogenesis-dominated (**B**), and methanogenesis plus methanotrophy (**C**) cyanobacterial mats. The schematic environment is supratidal or sabkha, where water depth is on the order of centimeters. Processes that have a minimal impact on isotope dynamics for each condition are rendered with low opacity to demonstrate that they are present even if they do not dominate isotopic signatures. Blue arrows represent fluxes with δ^13^C values approximately equivalent to local DIC. Green arrows represent fluxes with δ^13^C values approximately equivalent to local photosynthetically produced organic matter (25‰–30‰ below δ^13^C_DIC_). Orange arrows represent fluxes with δ^13^C values significantly lower than local photosynthetically produced organic matter (50‰ to >100‰ below δ^13^C_DIC_). The δ^13^C of atmospheric CO_2_ is fixed by exchange with open ocean DIC (not shown). The loss of CH_4_ with very low δ^13^C enriches the overall environment in ^13^C through time, producing high δ^13^C_carb_ values (**B and C**). A flux of O_2_ out of the microbial mat provides an approximate redox balance for the CH_4_ flux if nearly all organic carbon is remineralized and very little is buried. Predicted δ^13^C_org_ values in environments with methanotrophs (**C**) are highly variable because the organic carbon is a mix of organics from photosynthesis and highly ^13^C-depleted organics from methanotrophy. The δ^13^C_org_ values measured would be very sensitive to the relative proportions of these two sources. Oxidation of CH_4_ to DIC within the mat or water column would reduce the ^13^C enrichment of DIC for any of the models, a condition that may be present in open ocean and deep-water environments (not shown).

The remineralization of organic carbon to DIC and CH_4_ is a key aspect of the biological carbon cycle. When oxidants like O_2_, sulfate, nitrate, and redox-sensitive metals are abundant, organic carbon is mostly oxidized to CO_2_, which contributes to local DIC. When oxidants are sparse or the communities are unable to use them, fermentation and methanogenesis predominate (14). Oxidation of organic matter has a different isotopic effect on DIC than methanogenesis (11, 12, 15). The carbon isotopic composition of DIC produced by organic matter oxidation is similar to that of the original organic carbon and to the δ^13^C of DIC removed by primary productivity (Fig. 1A). Thus, oxidation of organic matter that was locally produced rarely leads to isotopic shifts in local DIC; even in cases of very high primary productivity, isotopic enrichments relative to oceanic DIC are typically less than 5‰–6‰ (3, 16). In contrast, remineralization through fermentation breaks larger organics into smaller ones. When CO_2_ and CH_4_ are produced, the CO_2_ is significantly enriched and the CH_4_ is significantly depleted in ^13^C relative to the original organic matter, with a difference of at least 40‰ (14). Specifically, CH_4_ released to the local environment has δ^13^C_CH4_ values at least 20‰ lower than the original organic matter. Because CH_4_ is a relatively insoluble gas, some of it escapes to the atmosphere from shallow or exposed environments (Fig. 1B). In contrast, the ^13^C-enriched CO_2_ becomes part of the local DIC pool. Thus, the net result of methanogenesis and loss of CH_4_ to the atmosphere is the evolution of local δ^13^C_DIC_ to higher values [Fig. 1B; (11, 12)]. The magnitude of the ^13^C enrichment depends on the specific methanogenic metabolisms active and the rate of CH_4_ loss relative to re-equilibration of local DIC with oceanic DIC and atmospheric CO_2_ (12, 14).

If organic matter remineralization style caused the LJE, a global mechanism is required for restricting extreme δ^13^C_DIC_ values in time and space. The evolution and ecological expansion of respiration metabolisms provide this mechanism, and evidence for their evolution is preserved in the genomes of extant organisms. The timing of gene origins and diversifications can be reconstructed using the phylogeny of genes from extant organisms in molecular clock models [e.g., reference (17)]. Molecular clock models require assumptions about the rates of evolution and calibration to the rock record, both of which are poorly constrained for microbial evolution in early Earth’s history. However, the models highlight intervals of rapid ecological expansion of key metabolisms through processes of gene evolution, duplication, and transfer among organisms. The origin of genes coding for a specific metabolism marks the first time a lineage of organisms can construct the necessary enzymes. However, metabolism does not necessarily become ecologically widespread until it provides a distinct advantage in diverse environmental conditions (18). When this happens, gene phylogenies tend to show numerous duplication and lateral gene transfer events (18). Thus, molecular clock models of organic remineralization metabolisms provide important insights into the ecological importance of these metabolisms before versus during or after the LJE interval.

#### Fermentation and CH_4_ metabolisms

Molecular clock models suggest that the last common ancestor of extant life was capable of fermentation and that methanogenesis emerged before 3 Ga [e.g., reference (19–21)]. Although modern methanogenesis in anaerobic environments is dominated by Archaea, methane production via fermentation was likely an early bacterial metabolism as well. Aerobic methanogenesis by bacteria is common in oxic seawater and lake water (22–25), including by *Cyanobacteria* (26). In these environments, methane is sometimes a byproduct of other metabolisms that transform organic molecules (24, 26). Given the antiquity of methanogenesis as a metabolism, it may have been a major energy source for diverse Bacteria as well as Archaea during Archean time.

Methanotrophy likely evolved prior to a negative excursion in δ^13^C_org_ at about 2.7 Ga (15, 27, 28). Thus, fermentation, methanogenesis, and methanotrophy were ecologically widespread metabolisms for processing organic carbon prior to, during, and after the LJE.

##### Sulfur

Molecular clock models suggest that sulfite reduction and sulfide oxidation genes (dsrAB) likely originated very early in the history of life (18). The origination of sulfate reduction genes (aprAB) is harder to date with molecular clocks, but results are consistent with an origin prior to 2.5 Ga (18). Interestingly, dsrAB and aprAB genes show significant diversification during the interval of atmospheric oxygenation and the LJE (18). Similarly, sox genes for sulfate-thiosulfate transformations originated at about 2.4 Ga, near the start of the LJE, although they diversified later (18). Thus, there is significant evidence for diversification and ecological expansion of sulfur redox metabolisms in the LJE interval. In addition, others have suggested that the ecological importance of sulfate reducers was highly variable during the LJE due to low seawater sulfate concentrations [e.g., reference (28)]. Thus, sulfur-based respiration rates were likely highly variable during the LJE, depending on the metabolic capabilities of the organisms present and the availability of sulfur in various oxidation states in local environments.

##### Nitrogen

Nitrate reduction likely evolved early in the history of life (29–31), but molecular clock models suggest that it was not a common metabolism until after the oxygenation of the atmosphere and surface oceans, possibly near 1.5 Ga (31). These models are consistent with a paucity of nitrate and availability of ammonia in anoxic seawater prior to 2.4 Ga, leading to little evolutionary pressure to use nitrate. The oxygenation of the atmosphere and surface oceans would have significantly increased those pressures, likely leading to the evolution of more effective and diverse nitrogen metabolisms (31, 32). For example, the annamox metabolism appears not to have evolved until the oxygenation of the atmosphere even though it is a strictly anaerobic process (33). Overall, nitrate reduction was likely an available metabolism during the LJE, with evidence that it became more efficient and ecologically widespread after this interval.

##### Oxygen

The evolutionary history of aerobic respiration and other oxygen-based metabolisms is complicated by extensive lateral gene transfer of oxidase and oxygenase genes among distantly related organisms in addition to the conversion of numerous enzymes and non-enzymatic proteins to oxidases (34–42). For example, the evolution of a specific group of oxygenases (Baeyer–Villiger monooxygenases) in *Chloroflexi, Actinobacteria,* and *Proteobacteria* is associated with at least 68 lateral gene transfers, the first of which occurred within the LJE interval (42). Cytochrome oxidases that interact directly with O_2_ are important for aerobic respiration and may have evolved in terrestrial environments in the ancestors of extant acidophilic Fe(II) oxidizers (within *Proteobacteria*) (38, 39); molecular clock models suggest they expanded to other organisms through extensive lateral gene transfer associated with the oxygenation of the atmosphere (38, 39), including within the LJE interval. Similarly, different oxidases essential to aerobic respiration were acquired by the *Cyanobacteria*, *Melainabacteria*, and *Sericytochromatia* after their divergence as clades, and oxidases continued to be exchanged through lateral gene transfer during the diversification of these lineages (35–37). Although the ages of these acquisitions are not well constrained, comparisons of organismal and gene phylogenies require that some lateral gene transfer events must have occurred prior to the diversification of oxygenic *Cyanobacteria,* whereas others occurred afterward (34–36), possibly during the LJE.

Antioxidant systems also evolved and expanded ecologically after the oxygenation of the atmosphere, including within *Cyanobacteria* (43). Others evolved in different lineages. For example, nickel superoxide dismutases, which convert 2O_2_^-^ into H_2_O_2_ and O_2_, likely originated in *Actinobacteria* and were then transferred to diverse other clades, including *Cyanobacteria*, although possible timings of such transfers were not reported (34). Molecular clock models for iron, manganese, and nickel superoxide dismutases in *Cyanobacteria* show that the relevant genes are polyphyletic and thus the result of multiple gene transfers (44). Results also suggest that all three classes of superoxide dismutases were present in some organisms during Paleoproterozoic time, although they may not have been widely available until after the LJE interval (44).

Overall, the timeline of evolution and diversification of various enzymes that interact with O_2_ is poorly constrained but often tied to the oxygenation of the atmosphere, either quantitatively or conceptually, with continued diversification and lateral gene transfer extending into Phanerozoic time.

##### Other metabolisms

There is growing evidence for the expansion of other metabolisms after the oxygenation of the atmosphere and during the LJE. For example, molybdenum-based enzymes (MopB group) use diverse substrates, many of which became significantly more abundant after the oxygenation of the atmosphere, likely leading to significant lateral gene transfer and diversification of these enzymes during the LJE interval (45). Similarly, while ancestral dimethyl sulfoxide reductase (DMSO) enzymes were likely present in the last common ancestor of life, some DMSO groups emerged and diversified within the LJE interval (45). Even some anaerobic metabolisms, such as anaerobic ammonium oxidation and anaerobic methane oxidation, appear to have diversified during the LJE interval (33, 46), reflecting the ecologic importance of extremes in oxidation state between organic-rich sediments and oxygenated waters and atmosphere.

### Methane solubility

If fermentation and methanogenesis were the major processes remineralizing organic carbon prior to and during the LJE, the fate of the resulting CH_4_ is critical to the resulting isotopic signatures. Gas solubility is governed by Henry’s Law: the concentration of the dissolved gas is the product of its partial pressure in the gas phase and its Henry solubility, which is a function of temperature, pressure, and water chemistry. In general, gas solubility decreases with increasing temperature in sedimentary environments. Solubility also decreases with decreasing pressure, so gases are less soluble in warm shallow water than in cold deep water.

For CH_4_, the Henry solubility constant at standard conditions in pure water is about 1.4 × 10^−5^ mol/m^3^ Pa, similar to that of O_2_ at 1.3 × 10^−5^ mol/m^3^ Pa (47). The Henry solubility constant for CO_2aq_ is more than an order of magnitude higher at 3.3 × 10^−4^ mol/m^3^ Pa. In addition, CO_2_ reacts with water to form HCO_3_^-^ and CO_3_^2-^, so the relative concentration of dissolved inorganic carbon (DIC = [CO_2aq_] + [H_2_CO_3_] + [HCO_3_^-^] + [CO_3_^2-^]) is much higher than for CH_4_ or O_2_ at the same gas partial pressure (47). At pH values high enough to precipitate carbonate minerals, most of the DIC is HCO_3_^-^ or CO_3_^2-^ (48). Thus, the solubility of CO_2_ produced by microbial communities in environments relevant to the LJE model is orders of magnitude higher than CH_4_ or O_2_ in the same environments.

Water composition has complicated effects on gas solubility (47). For gases that do not ionize in water, such as CH_4_ or O_2_, increasing salinity decreases solubility. However, salinity and pH complicate CO_2_ solubility due to the ion pairing of HCO_3_^-^ and CO_3_^2-^ with cations in the water. In general, DIC increases with both increasing salinity and increasing pH at a constant partial pressure of CO_2_ (48). Thus, evaporitic environments on ocean margins contain more DIC and less CH_4_ or O_2_ than open ocean water.

The composition of the atmosphere is also important for gas solubility because the equilibrium concentration of a dissolved gas depends on its partial pressure in the gas phase. When the atmosphere contains more CH_4_, more CH_4_ remains dissolved in local water where it can be consumed by methanotrophs or oxidized to DIC.

Overall, the solubility of CH_4_ is lowest under an oxygenated atmosphere in warm, saline, shallow water environments, including intertidal, supratidal, and sabkha environments. These environments also have the shortest diffusion path between microbial sources of CH_4_ and the atmosphere, and any wave mixing of these shallow waters with the atmosphere would increase the rate of CH_4_ loss. Thus, the loss of CH_4_ to the atmosphere was likely rapid from many of the environments represented by the ^13^C-enriched carbonates of the LJE. Therefore, microbial CH_4_ production and its subsequent loss to the atmosphere have the potential to cause the ^13^C enrichments observed during the LJE.

## RESULTS

### An explanatory metabolism evolution model for the LJE interval

Molecular clock data suggest that oxygenation of the atmosphere led to a significant expansion of microbial respiration metabolisms. When these evolutionary changes are interpreted within the context of carbon isotopic data from the rock record, an explanatory metabolic evolution model emerges, which predicts the key characteristics of the LJE. In the model, the LJE initiated an ecological expansion of cyanobacterial primary productivity in shallow water environments. Organic carbon remineralization at the beginning of the LJE was dominated by fermentation and methanogenesis, producing extreme enrichments in ^13^C in environments where CH_4_ was lost to the atmosphere. Through time, environmental selection led to the evolution and proliferation of respiration metabolisms. As these respiratory metabolisms spread, they displaced fermentation and methanogenesis in most environments, leading to the loss of the extreme ^13^C-enrichment characteristics of the LJE. Thus, the overall duration of the LJE was determined by the rate of ecologic expansion of respiration metabolisms that did not create large carbon isotopic fractionations.

#### Initiation of the LJE with atmospheric oxygenation

In the metabolism evolution model, the LJE was enabled by the oxygenation of the atmosphere and the formation of a stratospheric ozone layer between 2.45 and 2.3 Ga (49–51). An ozone layer significantly reduced atmospheric UVB and UVC transmission; its formation as a permanent feature provided protection from this ionizing radiation to terrestrial and shallow water environments exposed to direct sunlight (41). Although life was present in these environments prior to the LJE interval [e.g., references (52, 53)], the reduction of UVB and UVC exposure significantly reduced radiative damage, allowing substantial increases in cyanobacterial primary productivity. High primary productivity can shift local δ^13^C_carb_ to higher values (by up to 5‰–6‰) by preferentially removing ^12^C_DIC_ faster than DIC re-equilibrates with the atmosphere during the day (16). Thus, increased primary productivity in shallow marine intertidal, supratidal, and sabkha environments likely provided part of the isotopic shift defining the start of the LJE.

Cyanobacterial primary productivity still faced challenges early in the LJE. UVA is poorly absorbed by ozone, and it creates reactive oxygen species in the presence of O_2_ (45). In response, *Cyanobacteria* evolved new antioxidant systems (41, 43) and the pigment scytonemin (54). They also diversified into many of the lineages extant today based on both molecular clock models and microfossils in the rock record (41, 55, 56). These innovations likely led to increased photosynthetic efficiency and additional increases in primary productivity within the LJE interval.

#### Causes of high δ^13^C_DIC_

High primary productivity provides substantial organic matter for fermentation and methanogenesis. In the explanatory metabolism evolution model, high primary productivity during the LJE supported methanogenesis that produced highly ^13^C-depleted CH_4_ and ^13^C-enriched CO_2_. The CO_2_ remained dissolved in local water, contributing to ^13^C enrichment of DIC and thus carbonates, whereas CH_4_ was lost to the atmosphere (Fig. 1B). Any CH_4_ that reacted with O_2_ to form CO_2_ lowered the ^13^C enrichment of DIC. Thus, ^13^C enrichment of DIC was highly influenced by the solubility of CH_4_ in local water and the rate of its loss to the atmosphere. The solubility of CH_4_ is very low in warm, saline, shallow water. These environments also have short diffusion paths between microbial sources of CH_4_ and the atmosphere, making CH_4_ loss rapid relative to deeper marine environments. The model predicts that open ocean δ^13^C_DIC_ was not affected by CH_4_ loss due to higher gas solubility, a longer residence time allowing oxidation of CH_4_ to CO_2_, the high volume of water relative to primary productivity, and wave mixing that facilitated equilibrium between DIC and atmospheric CO_2_. Thus, open marine environments likely retained global marine δ^13^C_DIC_ values near 0‰ even if methanogenesis was an important organic remineralization process. In contrast, restricted environments with low water volumes, high DIC, and little wave mixing were furthest from carbon isotopic equilibrium. All these factors predict that warm, restricted environments with high pH, especially shallow marine supratidal and sabkha environments, developed the highest δ^13^C_DIC_ values during the early LJE when methanogenesis was a major remineralizing metabolism. These environments are also those most supersaturated with respect to carbonate minerals, providing a mechanism for preserving the very high δ^13^C_carb_ observed in the rock record [(3) and references therein (10)].

The isotopic composition of organic matter depended on both DIC and microbial metabolisms. In the explanatory metabolic evolution model for the LJE, the majority of organic matter was produced by cyanobacterial primary productivity, predicting a δ^13^C_org_ that is 25‰–30‰ lower than local δ^13^C_DIC_ and δ^13^C_carb_ [Fig. 1B; e.g., reference (1)]. However, in some cases, methanotrophy incorporated ^13^C-depleted CH_4_ into its biomass, and the resulting organic matter had a much lower δ^13^C_org_ value [Fig. 1C; (8, 14, 15)]. If methanotrophy produced some of the biomass, the isotopic difference between preserved organic matter and associated carbonate minerals may have been significantly greater than the typical 25‰–30‰ from photosynthesis, and the organic matter may not have been ^13^C-enriched even if local DIC was (5, 8, 9).

#### Termination of the LJE due to respiration

In the explanatory metabolic evolution model, the end of the LJE reflects the ecological expansion of aerobic and other forms of respiration to the extent that they outcompeted fermentation and methanogenesis as remineralization processes. This transition likely occurred at different times in different places, depending on the details of the oxidants and microbial communities present. Mat communities continued to emit CH_4_ in addition to O_2_, as they do today (11, 12, 26, 57), but through time, improved efficiency and more diversity of carbon-oxidizing metabolisms decreased the proportion of primary productivity remineralized by methanogenesis. Once the CO_2_ provided to local DIC had approximately the same isotopic composition as the CO_2_ removed during primary productivity, local δ^13^C_DIC_ did not evolve to values greater than those that can be produced by high primary productivity alone.

## DISCUSSION

The explanatory metabolic evolution model for the LJE integrates atmospheric oxygenation, microbial metabolisms, and evolutionary change to explain observed δ^13^C_carb_ in the context of genomic records of metabolic evolution. It provides testable predictions and addresses several outstanding problems with interpreting the LJE (3), including: (i) the timing of the LJE and its connection to the oxygenation of the atmosphere, (ii) the poorly defined beginning and end to the LJE, (iii) the environmental distribution of high δ^13^C_carb_ values, and (iv) an absence of evidence of enhanced organic carbon burial during the LJE. It predicts interdependencies among microbial evolution, ecology, and environmental conditions that worked together to produce both the geologic and genomic records.

### Atmosphere-LJE dynamics

The proposed model mechanistically ties the LJE to the oxygenation of the atmosphere. Thus, it requires an explanation for why methanogenesis did not produce carbonates with high δ^13^C_carb_ during Archean time even though methanogenesis is predicted to have been a major metabolism. One explanation is that primary productivity in shallow marine environments was low due to a high UVB and UVC flux. However, a paucity of highly ^13^C-enriched carbonates could also be due to the composition of the Archean atmosphere. Several models suggest that prior to about 2.45 Ga, the partial pressure of CH_4_ was orders of magnitude higher than after atmospheric oxygenation (58–60). An atmospheric CH_4_ partial pressure as high as 20 µbars was proposed as a minimum to allow the formation of observed mass-independent sulfur isotopic fractionations (61). At these high partial pressures, significantly more CH_4_ would have remained within Archean ecosystems where it could have been oxidized by locally produced O_2_. Without the loss of CH_4_ to the atmosphere, local DIC would not have become as ^13^C enriched in most environments. However, environments with an appropriate sink for CH_4_ could have produced high δ^13^C_carb_ values prior to the oxygenation of the atmosphere. Such environments could include particularly high-temperature or saline environments with low gas solubility or ecosystems with high methanotrophy consuming the CH_4_. Facies variations in δ^13^C_carb_ or lower than normal δ^13^C_org_ relative to δ^13^C_carb_ provide potential geological tests for these processes (6, 8, 16).

During the transition from an anoxic to oxygenated atmosphere, high δ^13^C_carb_ values are possible [e.g., reference (62)]. The explanatory metabolism evolution model predicts that high δ^13^C_carb_ values would be associated with low atmospheric CH_4_ and the presence of an ozone layer as documented by an absence of mass-independent sulfur isotopic signatures (49, 51, 60, 62, 63). Intervals of low atmospheric CH_4_ could be indicated by cooler temperatures, including glaciation, because it is a very potent greenhouse gas. Detailed comparisons of sulfur isotopic signatures, intervals of glaciation, and δ^13^C_carb_ can be used to test and refine the metabolic evolution model.

Even though the LJE is the result of atmospheric oxygenation, the metabolic evolution model does not predict that the LJE led to an increase in atmosphere O_2_. δ^13^C_carb_ is usually interpreted as reflecting oceanic δ^13^C_DIC_ in the context of mass balance between carbonate and organic carbon burial (1, 2). This interpretational framework requires substantial organic carbon burial to produce exceptionally high δ^13^C_carb_. Since organic carbon is reduced, redox balance requires the accumulation of an oxidant when organic matter is buried. The oxidant is typically interpreted to be atmospheric O_2_, and the organic burial model has led to a proposed “overshoot” to high atmospheric O_2_ during the LJE (3, 5, 9, 13, 50, 64, 65). However, if the metabolic evolution model can explain all ^13^C-enriched carbonate rocks, shallow water δ^13^C_carb_ values were decoupled from global atmospheric O_2_ concentrations. In fact, given the similar solubilities of O_2_ and CH_4_, their fluxes to the atmosphere from high δ^13^C_carb_ environments may have been balanced. Thus, the metabolism evolution model for the LJE does not require that global net organic matter burial increased, although the model also does not exclude increased burial. Similarly, it does not imply an increase in the partial pressure of O_2_ of Earth’s atmosphere during the LJE interval. The explanatory metabolism evolution model is consistent with any atmospheric composition with enough O_2_ to provide a sufficient ozone layer to allow high cyanobacterial primary productivity in shallow water and exposed environments.

### Heterogeneity in δ^13^C_carb_ and δ^13^C_org_

The explanatory metabolic evolution model for the LJE makes several predictions for δ^13^C_carb_ and δ^13^C_org_. First, high δ^13^C_carb_ values emerged from ecological responses to a newly oxygenated atmosphere and formation of an ozone layer. Specifically, high δ^13^C_carb_ values developed locally and were influenced by conditions that affected CH_4_ production and escape to the atmosphere, such as primary productivity, temperature, and physical mixing processes like waves and tides. These vary on such short time scales that it is unlikely that carbonate precipitation captured rises and falls in δ^13^C_DIC_ in any given section (16). Rather, the sensitivity of δ^13^C_DIC_ to small changes in the rates of both physical and biological processes predicts that δ^13^C_DIC_ and thus preserved δ^13^C_carb_ would have been highly variable (16).

Second, the sensitivity of δ^13^C_DIC_ to CH_4_ loss predicts highly heterogenous δ^13^C_carb_ across depositional environments. The model predicts the highest δ^13^C_carb_ values in exposed and shallowest water environments, which would have had the fastest CH_4_ loss to the atmosphere. Deeper environments would have had δ^13^C_DIC_ close to global marine values due to slower loss of CH_4_ and more water exchange with global DIC reservoirs. Even though shallow water environments were the most likely to produce high δ^13^C_carb_, specific deeper environments could have produced them if there was a significant sink of CH_4_ such as bubble nucleation, an oxic zone in a nearby part of the water column, or very high rates of methanotrophy. Significant methanotrophy would be recorded by particularly low δ^13^C_org_ values relative to δ^13^C_carb_, which can be observed in the rock record [Fig. 1C; (8, 9, 14, 15)]. Similarly, CH_4_ bubble formation might leave physical signatures in sedimentary rocks [e.g., references (8, 9)], and O_2_ oases in the water column might leave observable geochemical signatures [e.g., references (66–69)].

Third, the highest δ^13^C_carb_ values in the LJE are predicted soon after the oxygenation of the atmosphere and the formation of an ozone layer. Specifically, LJE conditions would be widespread as soon as cyanobacterial primary productivity significantly increased in previously UV-irradiated environments. At this time, the proportion of methanogenesis relative to carbon-oxidizing metabolisms would have been the highest across environments. With time, δ^13^C_carb_ would have declined as carbon-oxidizing metabolisms proliferated in diverse ecosystems. This predicted decline in high δ^13^C_carb_ can be tested as the ages and depositional environments of relevant carbonates become better characterized.

### Evolutionary end to the LJE

The colonization of shallow depositional environments under an oxygenated atmosphere represents a significant ecological change that drove evolutionary innovation. High cyanobacterial productivity in newly UV-protected environments produced co-located organic matter and diverse oxidants, which provided opportunities for evolutionary innovation of novel and more effective metabolisms (18, 41, 43). The diversification of numerous gene families has been tied to the oxygenation of the atmosphere and its aftermath using molecular clock models, but these calibrations are too imprecise to constrain the rates of evolution through the LJE. However, the explanatory metabolism evolution model couples the duration of the LJE to the time it took for aerobic and other respiration metabolisms to displace methanogenesis in most shallow water environments. Because ecological expansion was likely gradual and spatially heterogeneous, the model predicts that the end of the LJE interval was indistinct, with the loss of high δ^13^C_carb_ spatially and temporally variable across preserved sequences. The indistinct termination of the LJE can be tested in the geological record, whereas additional molecular clock models for the evolution of respiration metabolisms will provide insights into its duration.

An indistinct end for the LJE interval is consistent with the observed complexity of microbial ecosystems. Modern microbial communities are highly diverse with different dominant processes depending on niche structure and environmental interdependencies. For example, rare environments dominated by methanogenic carbon remineralization still exist as do those dominated by aerobic respiration and sulfate reduction. These produce different isotopic signatures on a heterogenous Earth. Similar heterogeneity was likely even more widespread as the biosphere first adjusted to a newly oxygenated atmosphere.

#### Ecological implications

If the explanatory LJE metabolism evolution model is correct, this interval of time represents the emergence of a new ecosystem structure. Prior to the LJE, remineralization of organic matter was performed predominantly through fermentation and methanogenesis. The main control on the spatial distribution of microbial metabolisms would have been the availability of bond types in local organic matter. Thus, the spatial structure of ecosystems prior to the oxygenation of the atmosphere was probably mostly controlled by the locations of primary productivity that provided molecules for fermentation. The subsequent evolution of respiration processes would have changed this structure. Respiration uses oxidants whose concentrations depend on the biogeochemical production of the oxidants, their consumption, and transport within ecosystems. In microbial mats, these processes create steep redox gradients, which lead to layered ecosystems with organisms using and tolerating O_2_ most abundant at the surface, with anaerobic organisms arranged in the subsurface based on O_2_ tolerance and the distributions of the oxidants they require [e.g., reference (70)]. This structure allows the efficient use of energy for organic carbon remineralization. It also creates highly reducing environments as little as a millimeter away from the ones containing O_2_. These very steep redox gradients allow the cross-diffusion of reduced and oxidized species across small spatial scales. This shift in ecosystem structure likely influenced the evolution of the organisms constructing the ecosystems, creating a system greater than the sum of its parts.

### Planetary microbiology

Interestingly, the explanatory metabolism evolution model for the LJE highlights connections among genome evolution, ecosystem structure, and Earth’s surface chemistry at the planetary scale. Earth’s atmosphere is well mixed, and oxygenation of the atmosphere was a global process. This planetary-scale change was a result of microbial metabolic production of O_2_, in the context of all the other geological and biogeochemical processes that influence fluxes of reductants and oxidants to Earth’s surface. In turn, the planet-wide accumulation of atmospheric O_2_ changed selection pressures for genome evolution, and the combined environmental and metabolic changes transformed ecosystem structures. New ecosystems and resulting changes in organic carbon remineralization reshaped carbon fluxes in Earth’s surface environments, thus influencing the dynamics of the global carbon cycle. More efficient organic matter remineralization released more nutrients that likely fueled additional primary productivity, increasing the rate of carbon turnover within the biosphere. A new dynamic equilibrium was likely reached within the carbon cycle by the end of the LJE, one that set the stage for ecosystem structures until the evolution of animals with their ability to prey on other organisms, creating an even faster turnover of carbon within communities [e.g., reference (71)].

These changes are also connected to other global-scale processes, including nutrient and metal biogeochemical cycles, that moderate environments for ecosystems. The interdependences of geological, ecological, biogeochemical, and genomic change have created an intricately habitable and inhabited planet.

## MATERIALS AND METHODS

This manuscript is part of a larger project to reevaluate biogeochemical changes through early Earth’s history as represented in both the geologic and genomic records. The explanatory metabolism evolution model presented here was developed to solve specific problems with the LJE posed by Prave et al. (10) and Hodgskiss et al. (3). It is informed by insights from decades of research on relevant rocks on Earth and Mars; modern microbial mat communities in the context of their environments and biogeochemistry; and science and technology studies with an emphasis on intersectional feminist theory applied to science as a generative process [e.g., reference (72)]. No new data were produced.

## EDITOR'S NOTE

This article was handled by Betül Kaçar, who acted as a Guest Editor in consultation with the Editor in Chief, Gemma Reguera.
